# Response: Commentary: Seeing the conflict: an attentional account of reasoning errors

**DOI:** 10.3389/fpsyg.2018.00024

**Published:** 2018-01-26

**Authors:** André Mata, Mário B. Ferreira

**Affiliations:** Faculdade de Psicologia, CICPSI, Universidade de Lisboa, Lisbon, Portugal

**Keywords:** intuition, reasoning, decision making, bias, conflict detection, attention

We welcome Frey et al.'s (2017a) thoughtful commentary to our work. In Mata et al. (2017), we used eye-tracking to measure the attention that problem-solvers pay to the premises of reasoning problems. Together with previous findings (Mata et al., 2014), that research suggests that rather than attributing reasoning errors solely to a failure to think properly about the problem, those errors might emerge earlier, from not paying sufficient attention to critical, conflict-relevant parts of the problem, thereby precluding its correct representation and comprehension (see also Evans, 1984, 1989, 1996). Indeed, correct responders were more likely than incorrect responders to pay attention to conflict-relevant premises, and they were better at discriminating between conflict and no-conflict problems.

The concern voiced by Frey et al. (2017a) is that a reading of our findings might suggest that incorrect responders are generally conflict-insensitive. That was not our point. Our research was not targeted at refuting (or even studying) implicit conflict detection (in our studies, the crucial comparisons include correct responses to conflict problems, which is not the case in conflict-detection studies). Our goal was to investigate the role of attention in reasoning. But of course, Frey et al. are correct in noting that our findings have implications for the question that is closest to their concern: whether incorrect reasoners are conflict-sensitive. In that regard, we will now revisit our research with an eye on this question.

At the mean/group level, even though there is a great deal of evidence pointing to conflict detection (e.g., De Neys et al., 2011; Stupple et al., 2013), we systematically fail to find evidence for incorrect responders' conflict sensitivity. This is the case using various measures, including attention [measured via eye-tracking, Mata et al., 2017, or change detection, Mata et al., 2014], performance ratings (Mata et al., 2013; Mata and Almeida, 2014, Study 3), and reaction time (Ferreira et al., 2016; for parallel findings from other labs, see Pennycook et al., 2012; Travers et al., 2016).

A more nuanced view is an individual-differences approach, whereby some, though not all, incorrect responders are conflict-sensitive (Pennycook et al., 2014, 2015; Mevel et al., 2015). But here too we find mixed results regarding whether most responders are conflict-sensitive or insensitive. Frey et al. (2017a) analyzed data from Study 2, where 51.9–55.6% of incorrect responders showed some degree of conflict sensitivity—far from a clear majority, but still a sizeable percentage. However, in Study 1, that percentage was lower: 33.3–44.4%^1^. In change-detection studies (Mata et al., 2014), where problem-solvers are asked whether they see any difference between the original conflict version of reasoning problems and slightly modified no-conflict versions of the same problems, conflict-sensitive incorrect responders are sometimes also the minority. And when care is taken to preclude detection of superficial changes (e.g., the conflict version is longer than the no-conflict version, which might enable change detection based on length and not content), that rate drops to 10.7%.

To be sure, we are not precluding the possibility of implicit conflict detection. It is an intriguing possibility, which parallels findings in other areas of cognition (e.g., Graf and Schacter, 1985), and is supported by an impressive amount of evidence. Moreover, we should note that considering only the percentage of conflict-sensitive incorrect responders provides a conservative measure, as correct responders are conflict-sensitive (Mata et al., 2014, 2017; Pennycook et al., 2015), and therefore the overall rate of conflict detection is higher than that described in studies focusing on error. What we are suggesting is that further work is needed to understand when/why incorrect responders show conflict sensitivity. We wish to offer two suggestions, concerning necessary preconditions for conflict detection.

First, one might explore the implications of our attentional account to conflict detection (we regard these approaches as complementary rather than competing): When reasoners do not pay attention to the conflict-relevant premises and therefore fail to understand the problem correctly, the reason why they err is not necessarily that they are unaware of logical principles, but rather because they cannot detect a conflict that has not been perceived/represented to begin with. Thus, logical intuitions cannot be triggered because they are not evoked by the problem as it is represented.

This might explain why, in conflict-detection studies, the confidence of incorrect responders is consistently high, sometimes as high as 85% or even 91.5% (where 100% means completely confident; De Neys et al., 2013; Johnson et al., 2016; see also Mata et al., 2013; Scherer et al., 2017). Responders who do not attend to, and therefore miscomprehend, the conflict-relevant premises answer conflict problems as if they contained no conflict. Future studies might integrate these two perspectives by studying the joint effect of attention/comprehension and conflict detection. We would not expect conflict detection for incorrect responders who miss the critical premises.

Second, rather than testing whether most responders are conflict-sensitive or insensitive, or testing the relative sensitivity/efficiency of conflict detection, one might impose strong tests on a conflict-detection model, and assume that conflict detection should only occur when conflicting thoughts are present. For instance, experts, who presumably do not share the same biased intuitions as laymen, should not show conflict detection (Svedholm-Häkkinen, 2015; Obersteiner et al., 2016). Conversely, young children, who have not yet accumulated the knowledge that underlies intuitive judgments should feel less conflicted (Jacobs and Potenza, 1991; Davidson, 1995; De Neys and Vanderputte, 2011).

In short, future research should test whether there is conflict detection only when the key ingredients are present: paying attention to the conflict-relevant premises, and having conflicting thoughts about the problem (e.g., for conjunction/base rate tasks, having knowledge of both the stereotype and the logical rule). As a noteworthy example, Frey et al. (2017b, Study 3) showed how differences in storage failure underlie conflict-sensitivity, and that only those incorrect responders who know the correct reasoning rules are conflict-sensitive. This is precisely the kind of approach that should bring valuable new insights to this debate.

## Author contributions

AM wrote the manuscript. MF provided comments.

### Conflict of interest statement

The authors declare that the research was conducted in the absence of any commercial or financial relationships that could be construed as a potential conflict of interest.

## Footnotes

^1^Frey et al. argued that Study 1 had a potential confound, such that critical elements of the reasoning process (reviewing of premises) were neglected. This was not the case: The question was presented together with the premises, and eye-tracking was continuously recorded while participants took their time to think about the problem before they decided to type their response. They could therefore freely re-read the initial premises in light of the question, as all the information was on display.
